# Therapeutic Plasma Exchange: A potential Management Strategy for Critically Ill COVID-19 Patients

**DOI:** 10.1177/0885066620940259

**Published:** 2020-07-15

**Authors:** Seena Tabibi, Tara Tabibi, Rosalynn R. Z. Conic, Nassim Banisaeed, Michael B. Streiff

**Affiliations:** 1 Department of Pathology, The Johns Hopkins University School of Medicine, Baltimore, MD, USA; 2 St. Louis University School of Medicine, St. Louis, MO, USA; 3 Department of Surgery, University of Maryland, Baltimore, MD, USA; 4 Southern Illinois University School of Medicine, Springfield, IL, USA; 5 Department of Medicine, The Johns Hopkins University School of Medicine, Baltimore, MD, USA

**Keywords:** plasma exchange, COVID-19, cytokine storm, ARDS, coagulopathy, DIC, endotheliopathy

## Abstract

In the 5 months since initial reports of COVID-19 came to light, the death toll due to SARS-CoV-2 has rapidly increased. The morbidity and mortality of the infection varies based upon patient age, comorbid conditions, viral load, and the availability of effective treatments. Findings from limited autopsies, clinical observations, and laboratory data suggest that high cytokine levels and a procoagulant state can precipitate acute respiratory distress syndrome and multi-organ dysfunction syndrome in critically ill patients. To complicate matters, comorbidities may affect the response to medical treatments currently in use, all of which are still in trial phase. Therapeutic plasma exchange (TPE) merits consideration in the treatment of critically ill COVID-19 patients and is an avenue for clinical trials to pursue. If efficacious, faster recovery of patients may lead to shorter intensive care unit stays and less time on mechanical ventilation. Herein, we briefly discuss some of the various approaches currently being investigated for the treatment of SARS-CoV-2 with a focus on potential benefits of TPE for selected critically ill patients.

## Background and Reasoning

The human impact of COVID-19 pandemic caused by SARS-CoV-2 is immeasurable. From health care to daily life to global economy, the pandemic has brought society to a grinding halt. At the time of writing, according to the Johns Hopkins University Coronavirus Resource Center, there are more than 1 840 000 positive cases globally, with over 113 000 patients succumbing to COVID-19.^1^

An estimated 5% to 10% of COVID-19 patients require intensive care unit (ICU) admission and mechanical ventilation.^2^ A study from Seattle, the first major US epicenter for COVID-19, analyzed 24 patients who were admitted to 9 Seattle area hospitals. Among the 12 survivors, median length of hospital stay, ICU stay, and mechanical ventilation were 17, 14, and 10 days, respectively.^3^ In one hospital in China, 17% of COVID-19 patients developed acute respiratory distress syndrome (ARDS).^4,5^ For the purpose of this article, critically ill patients are defined by either ICU admission and/or use of mechanical ventilation. COVID-19 patients have been managed using various therapeutic agents, all of which remain experimental. Therefore, prevention and containment of infection through public health measures have been the primary focus until effective treatments and vaccination become available. Some of the currently used therapies are briefly highlighted in the sections that follow.

### Direct Antiviral Effects

Remdesivir is an adenosine analog that can result in premature termination of viral replication, potentially leading to destruction of SARS-CoV-2.^6^ Clinical trials are actively investigating the role of Remdesivir in the treatment of COVID-19, given its broad-spectrum anti-CoV activity and promising efficacy through case reports.^7–9^

### Passive Immunity

Infusion of convalescent plasma aims to suppress SARS-CoV-2 viral load through transfer of neutralizing antibodies from donors who survived recent infection.^10^

### Modulation of Hyperimmune Responses

Hydroxychloroquine and chloroquine neutralize the acidic environment of cellular endosomes, hypothetically impairing the entrance of virus into host cells and halting its subsequent replication and virion assembly.^11^ By downregulating Toll-like receptor signaling and dampening production of inflammatory cytokines, hydroxychloroquine is also postulated to decrease the severity of COVID-19 symptoms.^12^ Finally, Tocilizumab, which binds to interleukin-6 (IL-6) receptor and inhibits IL-6 signaling, is currently under phase II clinical trials for COVID-19 patients in the United States.^13^ Analysis of patients in China found that patients with more extensive lung damage had elevated levels of IL-6.^14^

Yet, an important question is: Does morbidity and mortality of COVID-19 result primarily from immune response to the virus or from direct viral virulence?

While the exact answer is under investigation, immune responses, specifically cytokine storm and coagulopathy, play pathophysiologic roles in outcomes.^15–18^

Acute respiratory distress syndrome is the leading cause of mortality from COVID-19, and inflammatory mediators may contribute to ARDS.^16,19,20^ Minimally invasive autopsies performed on 3 COVID-19 patients confirmed the presence of ARDS.^21^ Findings included hyaline membranes, alveolar epithelial desquamation, alveolar hemorrhage, interstitial fibrosis, and chronic inflammation.^21^ After gaining entry into cells via the angiotensin-converting enzyme 2 receptor, SARS-CoV-2 may result in rapid epithelial and endothelial cell death and vascular damage.^22^ Other COVID-19 comorbidities include sepsis, septic shock, and disseminated intravascular coagulation (DIC), with multi-organ failure leading to mortality.^18,23^

Although current therapies have shown some promise, a complementary approach for critically ill patients with ARDS and multi-organ failure may be the use of therapeutic plasma exchange (TPE). In fact, TPE was proposed by Keith et al as a potential therapeutic measure for critically ill COVID-19 patients, targeting the complex effects of cytokine storm, inflammation, endothelial dysfunction, and coagulation dysfunction.^24^

Data suggest that TPE may have a role in clinical stabilization and recovery of critically ill COVID-19 patients.^25^ A recent case report described a 50-year-old woman with COVID-19 who had been admitted to a hospital after an 8-day history of intermittent dry cough, myalgia, fatigue, headache, and decreased appetite.^25^ She failed a trial of interferon (IFN)-α2B, lopinavir and ritonavir, and empiric ceftriaxone.^25^ She continued to deteriorate and required intravenous (IV) fluids, norepinephrine, empirical intravenous immunoglobulin (IVIG), and thymalfasin.^25^ She developed acute hypoxemic respiratory failure with increased ground-glass opacities and high-density consolidation on computed tomography imaging.^25^ At that time, TPE was initiated with 6 L of frozen plasma as the sole replacement fluid.^25^ After the third TPE session, she experienced resolution of respiratory symptoms and hypotension. After the fourth session, she experienced full recovery.^25^ Of note, she did not have any adverse side effects from TPE.^25^

The following subsections allude to information on potential applicability of TPE based on the role of cytokine storm and coagulopathy in pathophysiology of COVID-19.

#### COVID-19-mediated cytokine storm

A cytokine storm effect is postulated to lead to ARDS in COVID-19.^15,26,27^ Severe pneumonia caused by COVID-19 is associated with massive pulmonary inflammatory infiltrates and elevated serum levels of pro-inflammatory cytokines/chemokines.^15^ Wang et al studied 69 COVID-19 patients in Wuhan and found increased inflammatory cytokines among affected patients with a mortality rate of 7.5%.^28^ Patients with oxygen saturations (O_2_ sat) less than 90% had elevated levels of IL-6, IL-10, lactate dehydrogenase, and C-reactive protein (CRP).^28^ Additionally, Chen et al analyzed 29 patients with COVID-19 and classified them into mild, severe, and critical categories.^29^ The highest levels of IL-2R and IL-6 were seen in patients in the critical category.^29^ Interleukin-6 has been described as a key molecule in pathophysiology of cytokine storms.^30^ Moreover, patients in Wuhan, China, requiring ICU admission had higher levels of granulocyte colony–stimulating factor (G-CSF), IFN-γ-inducible protein 10, monocyte chemoattractant protein 1 (MCP1), macrophage inflammatory protein 1A, and tumor necrosis factor-α (TNF-α), suggesting that cytokine storm was associated with greater disease severity.^14^

The immune response in COVID-19-induced ARDS appears similar to those seen in macrophage activation syndrome (MAS) and secondary hemophagocytic lymphohistiocytosis (sHLH).^20,31^ McGonagle et al explored COVID-19 pulmonary immunopathology and described the potential benefits and disadvantages of IL-6 antagonism in patients with severe inflammatory responses.^31^ Clinical and laboratory manifestations of a MAS/sHLH picture seen in systemic onset juvenile inflammatory arthritis were compared to potential MAS complications with COVID-19-infected patients. Not only highly elevated CRP and ferritin, which are key to the diagnosis of MAS/sHLH, are also elevated in many severe COVID-19 pneumonia cases, the cytokine profile in MAS/sHLH resembles that of COVID-19 patients, with increased levels of IL-1β, IL-2, IL-6, IL-17, IL-8, TNF, and MCP1.^31–36^ This has generated tremendous interest in anti-cytokine strategies aimed at dampening exaggerated immune responses.^31,37–40^

There is inherent complexity in targeting multiple cytokines via pharmaceutical agents. Therapeutic plasma exchange may alleviate the need for multiple medications against various cytokines, avoiding the side effects from polypharmacy and the need for dose adjustments in patients with comorbidities.

In one study, Luo et al examined various parameters including CRP and IL-6 levels of 6 critically ill COVID-19 patients.^41^ All patients had evidence of respiratory dysfunction and 3 of the patients underwent plasma exchange.^41^ Both CRP and IL-6 levels were elevated in these patients prior to TPE.^41^ After plasma exchange, CRP and IL-6 levels decreased in all 3 patients.^41^ With regard to IL-6 levels specifically, prior to plasma exchange, the 3 patients had IL-6 values of 12.14, 12.20, and 142.90 pg/L, respectively.^41^ After plasma exchange, IL-6 values decreased to 4.33, 2.55, and 6.48 pg/L, respectively.^41^ Currently, although there are no formal guidelines regarding the exact IL-6 value above which plasma exchange should be utilized, further exploration of the relationship between IL-6 levels in critically ill COVID-19 patients and the impact of plasma exchange on these values may prove to be invaluable.

Therapeutic plasma exchange has also been previously used in the treatment of ARDS during the 2009 global pandemic of H1N1 influenza.^42^ Three children in pediatric ICU had developed ARDS, cytokine release storm (CRS), and were hemodynamically compromised.^42^ All 3 were placed on mechanical ventilation with nitric oxide, and 1 patient was also on extracorporeal membrane oxygenation.^42^ As a last resort, TPE was performed to mitigate the patients’ CRS.^42^ Using the filtration exchange method, the patients underwent 2 TPE procedures, which lead to dramatic reductions in oxygen and vasopressor requirements and significant drops in their pediatric organ dysfunction scores.^42^ All 3 patients tolerated TPE procedures without any adverse effects and ultimately survived with a good functional status at discharge.^42^ In another study, TPE was used to treat ARDS in a 41-year-old man with a history of myasthenia gravis in remission, who had developed ARDS secondary to pneumonia.^43^ When his oxygenation continued to deteriorate despite invasive mechanical ventilation, nanomembrane-based apheresis was employed, and after 3 sessions, the patient was successfully weaned off the ventilator.^43^

Another condition where TPE has been used successfully in reducing markedly elevated cytokines is IVIG-resistant Kawasaki disease (KD), a vasculitic, coagulopathic condition associated with coronary artery aneurysms and myocardial ischemia.^44–49^ Elevated inflammatory cytokines including TNF-α, IL-1β, IL-6, IL-8, and IFN-γ have been reported in the acute phase of KD.^44^ Fujimaru et al demonstrated that TPE can remove circulating inflammatory cytokines in these patients.^44^ Specifically, plasma levels of multiple key pathophysiologic cytokines and inflammatory mediators (IL-6, IL-8, IL-10, TNF-α, IL-17, G-CSF, sTNFR1, and sTNFR2) were decreased after TPE.^44^ The IL-6 levels decreased from 105.1 pg/mL on day 0 to 17 pg/mL on days 4 to 5 following plasma exchange (*P* = .02).^44^ In other examples, IL-8 levels decreased from 21.2 pg/mL on day 0 to 14.1 pg/mL on days 4 to 5 after plasma exchange (*P* = .04) and IL-17 levels decreased from 20.9 pg/mL on day 0 to 6.2 pg/mL on days 4 to 5 after plasma exchange (*P* = .02).^44^ In a separate case report by Kashiwagi et al, a 2-year-old patient with KD and elevated IL-6 levels refractory to IVIG was successfully treated by TPE, without procedural adverse effects.^45^

Therapeutic plasma exchange has been reported to reduce key pro-inflammatory cytokines in patients with septic shock.^24,46^ Knaup et al used a prospective single-center, open-label, nonrandomized pilot study to investigate the role of TPE in patients with early septic shock (onset less than 12 hours) who required high doses of norepinephrine.^46^ Not only was TPE well tolerated without any adverse events, but it also reduced key pro-inflammatory cytokines and a key permeability factor, including IL-6, IL-1β, and angiopoietin-2.^46^

On April 10, 2020, the US Food and Drug Administration (FDA) gave emergency use authorization for

Spectra Optia Apheresis System with the Depuro D2000 Adsorption Cartridge to treat patients 18 years of age or older with confirmed COVID-19 admitted to the ICU with confirmed or imminent respiratory failure by reducing pro-inflammatory cytokine levels which may ameliorate a cytokine storm due to the overabundance of pro-inflammatory cytokines and, in turn, provide clinical benefit to such patients.^50^

Given the diverse application of TPE in conditions marked by hypercytokinemia, utilization of TPE in the current pandemic can open the doors to a new approach in treatment of COVID-19.

#### COVID-19-mediated coagulopathy

Alongside cytokine-mediated lung injury, coagulopathy appears to play a role in the demise of COVID-19 patients.^18^ COVID-19 patients who succumbed to their illness had significantly elevated fibrinogen degradation products and d-dimer levels compared to patients with mild disease.^18,51^ Wang et al who studied 138 COVID-19 patients found d-dimer of ICU versus non-ICU patients were 414 and 166 μg/L, respectively.^52^ They also reported that d-dimer levels were higher in nonsurvivors than survivors.^52^ d-Dimer levels above 1000 μg/L may identify COVID-19 patients with poor prognosis at an early stage.^53^ A meta-analysis of 1779 COVID-19 patients also suggested thrombocytopenia as a clinical marker of severe disease.^18,54,55^ These findings are in line with the consumptive coagulopathy that patients with COVID-19 may experience.^56^ Approximately 80% of critically ill COVID-19 patients on mechanical ventilation in one hospital in China had evidence of DIC.^18^ Computed tomography imaging and autopsy findings in COVID-19 patients have also described vascular changes, including vascular thickening and microvascular pulmonary thrombosis.^21,57–61^ Potential mechanisms leading to coagulopathy in COVID-19 patients may include direct viral damage, cytokine storm, platelet aggregation and thrombosis in lungs along with increased platelet consumption and destruction, and potential underlying hypercoagulability in critically ill patients.^18,62^

Pulmonary endotheliopathy has been a long known key factor in ARDS, resulting in inflammation and microthrombosis.^56,63^ While the inflammatory pathway is triggered by the release of inflammatory cytokines, the microthrombotic pathway results from exocytosis of unusually large von Willebrand factor multimers (ULVWF multimers) and platelet activation.^56^ The platelet-ULVWF complexes then deposit on injured endothelial cells and result in endotheliopathy-associated vascular microthrombotic disease (EA-VMTD), leading to consumptive thrombocytopenia, multi-organ dysfunction syndrome (MODS), and DIC.^56^ Acute respiratory distress syndrome can thus be viewed as one of many end-organ phenotypes among MODS associated with underlying EA-VMTD and thrombotic thrombocytopenic purpura (TTP)-like syndrome.^56^

If coagulopathy is not addressed in thrombotic microangiopathy (TMA), the mortality risk is high.^64^ Several studies have analyzed the potential role of TPE for patients with secondary TMA.^64^ A retrospective study of 76 patients by Stegmayr et al reported that adults with DIC and multi-organ failure who received plasma exchange had an 82% survival rate whereas less than 20% survival was observed among historical controls.^64,65^ Along a similar theme, Pene et al found that regardless of disease severity, in adults when TMA was triggered by infections, the use of plasma exchange is independently associated with lower mortality (hazard ratio = 0.234; 95% CI, 0.095-0.573).^64,66^

Therapeutic plasma exchange has been fundamental in reducing mortality (from 80% to 20%) and improving clinical outcomes in TTP.^48,67^ According to the 2019 Guidelines from the American Society for Apheresis, TPE has a category I indication with grade 1A recommendation in treatment of TTP, meaning that it is accepted as first-line therapy, either alone or in conjunction with other modes of treatment.^48^ In addition to removal of a disintegrin and metalloproteinase with a thrombospondin type 1 motif, member 13 (ADAMTS13) autoantibodies, the primary mechanism for TPE in TTP is through plasma replenishment of ADAMTS13, the protease that cleaves ULVWF multimers.^48,56^

Multiple studies have shown that systemic inflammation in the setting of sepsis, especially due to an infectious etiology, can result in acquired deficiency of ADAMTS13.^68–70^ A study showed that platelets were markedly reduced in patients with ADAMTS13 activity <30% (39 000/μL) versus ADAMTS13 activity ≥ 30% (193 000/μL, *P* = .004). The acquired ADAMTS13 deficiency in such settings can exacerbate the levels of ULVWF multimers that may already be increased from EA-VMTD and TTP-like syndrome in ARDS and MODS.^56,70^ Decreased ADAMTS13 levels correlate with progression to multi-organ failure and, along with appearance of ULVWF multimers in plasma, have been associated with an increased risk of mortality in patients with sepsis.^69,70^

Stahl et al reported that TPE using plasma from healthy donors increased the activity of antithrombin-III (ATIII) and protein C, which were markedly reduced in patients with sepsis (pre-TPE ATIII activity: 51% vs post-TPE ATIII activity: 63%, *P* = .029; pre-TPE protein C activity 47% vs post-TPE protein C activity: 62%, *P* = .029).^68^ In the same study, prior to TPE, ADAMTS13 activity was markedly reduced while von Willebrand factor antigen (vWF: Ag) levels were markedly elevated.^68^ Therapeutic plasma exchange was able to significantly increase ADAMTS13 levels while reducing vWF: Ag levels (pre-TPE median ADAMTS13 activity: 27%, post-TPE median ADAMTS13 activity: 47%, *P* < .001; pre-TPE vWF: Ag: 353 IU/dL, post-TPE vWF: Ag: 170 IU/dL, *P* < .001).^68^ Therapeutic plasma exchange could potentially remove activated procoagulant proteins while replacing natural anticoagulants using donor plasma.^68^

More data regarding endotheliopathy-mediated pathways specific to COVID-19 patients are needed. At the same time, given that critically ill COVID-19 patients may be succumbing to processes in which endotheliopathy is a core pathophysiologic feature, TPE may have a therapeutic role. Clinical trials can more definitively explore TPE’s role in these patients.

## Safety, Suggestions, and Considerations for Use of TPE

Since the target population of TPE use are critically ill COVID-19 patients, safety of TPE is of crucial importance in ICU patients. A study of ICU patients who received TPE for a range of indications found the following list as the most frequent adverse side effects: decreased arterial blood pressure (8.4% of procedures), arrhythmias (3.5% of procedures), paresthesia (1.1% of procedures), and cold sensation with transient increases in body temperature (1.1% of procedures).^71^ Severe and life-threatening symptoms such as shock, decrease in blood pressure requiring vasopressors, persistent arrhythmia, and hemolysis developed in 2.16% of all procedures performed in ICU patients.^71^ The authors concluded that TPE is a safe procedure for ICU patients.^71^

In a retrospective study by Ataca et al comparing TPE procedures performed on 981 geriatric patients and 3728 nongeriatric patients, the most common indications for TPE in both groups were sepsis/ARDS and multiple organ dysfunction.^72^ Of note, the study found that the complication rate was statistically similar between both groups and that when performed by experienced staff, TPE was a safe procedure for geriatric patients.^72^

In addition to the case reports presented earlier in the manuscript, plasma exchange has already been used safely as a therapeutic modality during the current COVID-19 pandemic.^73^ Preliminary data from a retrospective study by Tian et al in China mentioned the use of plasma exchange in critically ill patients with moderate ARDS.^73^ This study, which focused on 37 COVID-19 patients outside Hubei Province in China, primarily analyzed patients with mild-to-moderate disease and did not go significantly in depth on the efficacy of the different range of therapies used in their patients as it was primarily an observational study.^73^ Nevertheless, the use of plasma exchange resulted in no serious adverse events such as shock, pulmonary embolism, renal injury, or DIC, and patients recovered from COVID-19.^73^ Overall, TPE, which has been performed for over a century, is shown to be safe and efficacious in several disorders.^74^ Also, acquiring SARS-CoV-2 through blood products has not been reported to this date.^75^

One concern regarding TPE could be negative impact on blood supplies, which indeed have become valuable commodities during the pandemic.^75,76^ Blood donations in Washington State, United States’ first epicenter for COVID-19, dropped significantly during the first week of March—just as the pandemic had started to affect the state.^76^ However, with more diligent triaging of blood orders, prioritizing blood utilization, and postponing elective procedures at a hospital-based transfusion service, blood supply inventories were able to meet demands by the second week of March.^76^ Although addressing blood donation shortages may not be completely achievable in every center at any given time, there are avenues to mitigate these shortages.^76^

As an alternative, some TPE procedures can be completed by 5% albumin as the replacement fluid; however, critically ill COVID-19 patients have a high incidence of coagulopathy and TPE procedures with the use of albumin may result in depletion of procoagulant factors and increased bleeding risk.^18,47,77,78^ The time required for these factors to regain their original concentrations varies and can take up to 18 days, depending on the substance.^47,78^ This may necessitate the use of plasma as the replacement fluid. Nevertheless, the risk of clinically significant bleeding with plasma may not be high: A meta-analysis of anticoagulated patients who underwent TPE did not appear to show significant increase in bleeding or thrombotic risk.^79^

An important consideration is to develop criteria for the best time for TPE intervention in critically ill patients. Deng et al analyzed the clinical characteristics of survivors versus nonsurvivors of COVID-19 in Wuhan, China, and found that 89.9% of nonsurvivors versus only 7.6% of survivors had ARDS (χ^2^ = 148.105, *P* < .001).^80^ Their findings correlated well with O_2_ sat levels: Nonsurvivors of COVID-19 had significantly lower O_2_ sat levels compared to survivors (nonsurvivors’ O_2_ sat: 85% [range: 77%-91%]; survivors’ O_2_ sat: 97% [range: 95%-98%]; *Z* = 10.625, *P* < .001).^80^ Another striking finding is that the lowest range of O_2_ sat range in survivors (95%) did not overlap with the highest range of O_2_ sat among nonsurvivors (91%).^80^ Patients with ARDS may respond to TPE if used early in the course of illness.^56^ It would, therefore, be beneficial to identify candidates early on, prior to further deterioration.^56^ Following this theme and in order to maintain consistent definitions, TPE can be employed based on the same criteria that are recommended for investigational COVID-19 convalescent plasma and for FDA’s recent approval for Spectra Optia Apheresis System from the National Expanded Access Treatment Protocol for COVID-19^50,81^:

1. Early acute lung injury/early ARDS; or
2. Severe disease, defined as:
  - Dyspnea
  - Respiratory frequency ≥30/minute
  - Blood O_2_ sat ≤93%
  - Partial pressure of arterial O_2_ to fraction of inspired O_2_ <300
  - Lung infiltrates >50% within 24 to 48 hours
3. Life-threatening disease, defined as:
  - Respiratory failure
  - Septic shock, and/or
  - Multiple organ dysfunction or failure

One specific logistic aspect for utilization of TPE is the cost, which varies. For example, in the United States, disposables for apheresis cost between US$1500 and US$3000 per patient excluding other costs including the cost of trained professionals such as nurses.^67^ However, in order to accurately evaluate cost efficiency, it is crucial to weigh other factors. A methodological review of expenses in *Critical Care Medicine* within the United States found that in 2010, the cost of 1-day stay at the ICU was approximately US$4300.^82^ Though this value may not reflect current ICU expenses, approximately 5% to 10% of COVID-19 patients require ICU admission.^2^ Given that the median ICU stay in survivors from one study based in the Seattle area was 14 days, the costs of care per each ICU patient are tremendous.^2,3^ If TPE successfully decreases the median time of ICU stay and/or median time on mechanical ventilation by even a few days, the overall savings could be substantial and the procedure may be deemed cost-efficient.

Other considerations include availability and performance time of the procedure. Apheresis availability varies within the United States and globally and may not be readily available in many locations.^83^ Furthermore, TPE requires skilled staff with advanced expertise.^84^ Patients require monitoring during the entirety of the procedure (approximately 2 hours) with temporal assessment of vital signs and clinical symptoms should any arise. Last but not least, performing staff will be in relatively close proximity to COVID-19 patients, although personal protective equipment worn at all times will reduce the risks of exposure.

## Conclusion

Although public health measures to limit viral spread should continue to be in place, it is essential to identify effective therapeutic modalities for the treatment of COVID-19. Medical therapeutics under current investigation may ultimately provide effective early interventions for COVID-19; however, TPE merits investigation as a means to stabilize critically ill or rapidly deteriorating patients to reduce mortality. Despite concerns regarding availability, the new emergency use approval by FDA merits clinical trials to assess the role of TPE in critically ill COVID-19 patients. In addition to its overall safety, clinical trials evaluating TPE use in COVID-19-induced ARDS and multi-organ dysfunction could open doors to further explore TPE as an adjunctive treatment strategy in the management of future respiratory viral pandemics resulting in ARDS and vasculopathy.
